# Lateral Spacing of TiO_2_ Nanotubes Modulates Osteoblast Behavior

**DOI:** 10.3390/ma12182956

**Published:** 2019-09-12

**Authors:** Madalina Georgiana Necula, Anca Mazare, Raluca Nicoleta Ion, Selda Ozkan, Jung Park, Patrik Schmuki, Anisoara Cimpean

**Affiliations:** 1 Department of Biochemistry and Molecular Biology, University of Bucharest, 050095 Bucharest, Romania; necula.madalina92@gmail.com (M.G.N.); rciubar@yahoo.com (R.N.I.); 2 Department of Materials Science WW4-LKO, Friedrich-Alexander University, 91058 Erlangen, Germany; anca.mazare@fau.de (A.M.); selda.oezkan@ww.uni-erlangen.de (S.O.); schmuki@ww.uni-erlangen.de (P.S.); 3 Division of Molecular Pediatrics, Department of Pediatrics, University Hospital Erlangen, 91054 Erlangen, Germany; jung.park@uk-erlangen.de

**Keywords:** spaced TiO_2_ nanotubes, osteoblast, cell adhesion and morphology, cell proliferation, osteogenic differentiation

## Abstract

Titanium dioxide (TiO_2_) nanotube coated substrates have revolutionized the concept of implant in a number of ways, being endowed with superior osseointegration properties and local drug delivery capacity. While accumulating reports describe the influence of nanotube diameter on cell behavior, little is known about the effects of nanotube lateral spacing on cells involved in bone regeneration. In this context, in the present study the MC3T3-E1 murine pre-osteoblast cells behavior has been investigated by using TiO_2_ nanotubes of ~78 nm diameter and lateral spacing of 18 nm and 80 nm, respectively. Both nanostructured surfaces supported cell viability and proliferation in approximately equal extent. However, obvious differences in the cell spreading areas, morphologies, the organization of the actin cytoskeleton and the pattern of the focal adhesions were noticed. Furthermore, investigation of the pre-osteoblast differentiation potential indicated a higher capacity of larger spacing nanostructure to enhance the expression of the alkaline phosphatase, osteopontin and osteocalcin osteoblast specific markers inducing osteogenic differentiation. These findings provide the proof that lateral spacing of the TiO_2_ nanotube coated titanium (Ti) surfaces has to be considered in designing bone implants with improved biological performance.

## 1. Introduction

A key aspect for orthopedic implant integration is the ability to enhance the functional activity of osteoblasts at the tissue/implant interface without generating a fibrous tissue [1]. Despite its remarkable success as a bone tissue implant, accumulated experience in titanium (Ti) implantation has emphasized some aspects that need to be improved, such as the ability to stimulate osteointegration [2]. Therefore, research efforts have been focused on improving cell-material interactions, shown to depend on the surface physico-chemical properties of the implant and extensive studies have been made on evaluating these factors (wettability, roughness, topography) [3,4,5].

The addition of nanotopographic features to the surface of bone implants has become a growing area of research in the bone regenerative medicine, due to the nanometer scale structural hierarchy of natural bone [6]. Numerous studies emphasized the role of nanotopographical cues in directing osteoprogenitor cells behavior [7,8,9,10,11]. A simple, economical and easy method to modify the topography of Ti-based materials, widely used in orthopedics, is electrochemical anodization [12]. This process leads to the formation of self-ordered TiO_2_ nanostructures directly on the surface of the Ti substrate and more importantly, the morphology of anodic TiO_2_ nanotubes (TNTs), such as diameter, wall thickness and length, can be easily controlled through the anodization parameters (applied voltage, time, electrolyte compositions, temperature, etc.) [12,13]. Nowadays, a wide range of nanotubular structures can be grown such as smooth wall, stacks, single wall or spaced nanotubes [12,14].

Typically, anodic nanotubes grow in a closed packed hexagonal arrangement, showing very little or no spacing in between the nanotubes. Over the years, spaced or loose-packed nanotubes have been reported to grow in electrolytes containing diethylene glycol (DEG), dimethylsulfoxide (DMSO) or trietylene glycol electrolytes containing fluorine [14,15,16,17], or in very specific anodization condition (e.g., ethylene glycol, tri (tetra, poly)-ethylene glycol) [18,19].

Ti surfaces modified with anodic TNTs represent a highly biocompatible material that integrates well into the bone tissue, exhibits thermal and chemical stability, controllable dimensions, large contact surface, adjustable size pores, adjustable surface chemistry and high surface/volume ratio [4,12,20,21]. These particularities have proved to favor the proliferation and differentiation of bone cells [4,22].

Cell attachment and interaction with TNTs coated surfaces has been intensively studied, mostly in view of the effect of nanotube diameter on bone regeneration process [7,23,24,25,26,27], while other nanotube morphological parameters are less investigated, e.g., spacing. The impact of TNTs with different diameters varying from 15 nm to 100 nm on mesenchymal stem cells (MSCs) was initially reported by Park et al. [7,23], observing that 15 nm diameter nanotubes favor osteogenic differentiation by improving cell adhesion. At the same time, nanotubes with a diameter above 50 nm induced a sensitive alteration of stem cell behavior in terms of spreading and adhesion resulting in the reduction of cell proliferation, migration and differentiation and, finally, apoptosis. When the experimental conditions were optimized for cell-biomaterial interactions, additional cell proliferation and migration tests with osteoblasts, osteoclasts and endothelial cells showed similar results [12]. 

In case of the stem cells induced toward the committed status under high level of serum supplement or the already committed pre-osteoblast cell line, studies concluded that also a larger diameter from 70 nm to 100 nm can stimulate the growth and differentiation of bone cells [24,25,28,29]. Though the range of nanotubular diameters explored in various studies was confined up to 100 nm, there are also studies investigating osteoprogenitor cells interactions with large diameter TNTs (100 nm up to 470 nm) [30,31], indicating that 170 nm provides the optimal diameter to sustain osteoblast proliferation, differentiation and mineralization [31]. 

Regarding the spacing between the nanotubes, some studies hypothesized that this surface characteristic can contribute to the continuous flow of the culture medium as well as to the exchange of gases, nutrients and signaling molecules, thus stimulating the cellular metabolism [27,32] and mimicking better the in vivo conditions. Moreover, recent works have demonstrated that the cellular behavior can be positively or negatively regulated by the spacing between different nanostructures, such as nanorods [33,34] or nanopillars [35,36]. For example, Zhou et al. [33,34] synthesized strontium-doped hydroxyapatite (Sr_1_-HA) nanorods with different interrod spacing (67.3 ± 3.8, 95.7 ± 4.2, and 136.8 ± 8.7 nm) on microporous TiO_2_ and showed that the osteoblast adhesion, proliferation and differentiation can be regulated by the interrod spacing: the cellular response was significantly enhanced on the nanorods with spacing smaller than 96 nm while larger spacing exerted inhibitory effects. 

Given the fact that the biological effect generated by the spacing of nanotubular structures is not well known, this study aims to test the influence of TNT with a spacing of 80 nm (TNT80) or 18 nm (TNT18), and both with similar nanotube diameter of ~78 nm, on the MC3T3 pre-osteoblast response (as reference material in these studies, flat Ti surface was used). The choice of ~78 nm diameter is motivated by previous studies showing an optimal osteogenic differentiation on similar diameter nanotubes [29,30,31], in vivo studies evaluating the osseointegration capacity of TNT modified Ti implants showing that the implants coated with ~70 nm diameter TNTs induced an accelerated bone formation [29,37]. Lastly, our previous in vitro studies revealed that ~78 nm diameter TNTs mitigate the macrophages inflammatory response [38,39].

## 2. Materials and Methods 

### 2.1. TiO_2_ Nanotube Growth and Characterization

#### 2.1.1. TiO_2_ Nanotube Growth

To grow nanotubes, 0.125 mm thick Ti foil (99.6% pure temper annealed, ADVENT, Oxford, UK) was used, cut in 2.5 × 2.5 cm. Ti foils were cleaned by ultrasonication (Emmi-H30, EMAG AG, Germany) in acetone (Emsure Merck, Darmstadt, Germany), in ethanol (>99.8% p.a. Roth, Karlsruhe, Germany), followed by rinsing with distilled water and drying in a N_2_) stream. For anodization, a two-electrode configuration was used in which Pt (ADVENT, Oxford, UK) acts as a cathode, and the Ti as anode using an O-ring cell with a diameter of 2 cm. 

For TNT18 (nanotubes with ~78 nm diameter and 18 nm spacing at the top), the Ti foils were used as such and anodization was performed in Glycerol (>99.7% p.a. Roth, Karlsruhe, Germany): H_2_O (70:30 vol.%) + 0.5 wt.% Ammonium fluoride (NH_4_F, >98% p.a. Roth, Karlsruhe, Germany ) at 20 V for 2 h (room temperature). The distance between the electrodes was of 1.5 cm and around 50 mL of electrolyte were used.

The synthesis of spaced NTs with similar diameter and a spacing of 80 nm (TNT80) consists of a two-step anodization process. In the first step, the Ti foil was anodized in Ethylene glycol (>99.5% p.a. Roth, Karlsruhe, Germany) + 0.1 M NH_4_F + 1 M H_2_O at 53 V for 1 h, and then the nanotube layer was removed by ultrasonication in deionized water. The prepatterned substrate was used as substrate for the second anodization which was performed in Diethylene glycol (>99.5% p.a. Roth, Karlsruhe, Germany) + 4 wt.% Hydrofluoric acid (HF 40%, Sigma Aldrich, Germany) + 0.3 wt.% NH_4_F + 7 wt.% H_2_O at 27 V for 4h at 30 °C (using 60 mL electrolyte and 2 cm distance in between the electrodes). After anodization, spaced nanotubes were immersed in ethanol for 10 min, rinsed with distilled water and dried in an N_2_ stream.

#### 2.1.2. TiO_2_ Nanotube Characterization

Samples morphology was characterized by scanning electron microscope (SEM, FE-SEM 4800SEM, Hitachi, Japan) coupled with an energy-dispersive X-ray Spectroscope (EDAX Genesis, fitted to the SEM chamber), while their chemical composition and chemical state was investigated by using X-ray photoelectron spectroscopy (XPS, PHI 5600, Physical Electronics, US)—peaks were shifted to C1s 284.8eV.

### 2.2. Cell Culture

Experiments were performed using MC3T3-E1 murine pre-osteoblast cell line (ATCC^®^, CRL-2593TM) which was grown in Dulbecco’s Minimal Essential Medium (DMEM, Sigma-Aldrich Co.St. Louis, MO, USA) supplemented with 10% fetal bovine serum (FBS, Gibco, Grand Island, NY, USA) and 1% (*v/v*) penicillin/streptomycin (10,000 units mL^−1^ penicillin and 10 mg mL^−1^ streptomycin) (Sigma-Aldrich Co. St. Louis, MO, USA) in an incubator at 37 °C in humidified atmosphere with 5% CO_2_. These cells were seeded on the Ti, TNT18 and TN80 surfaces at an initial cell density of 1 × 10^4^ cells/cm^2^ to assess the cellular survival/proliferation, adhesion and morphology and maintained in standard culture conditions for up to three days. For pre-osteoblast differentiation studies, the cell seeding density was 4 × 10^4^ cells/cm^2^. These studies were performed under osteogenic conditions by supplementing the standard culture medium with 50 μg/mL ascorbic acid (Sigma-Aldrich Co.St. Louis, MO, USA), 5 mM β-glycerophosphate (Sigma-Aldrich Co. St. Louis, MO, USA) and 10^−8^ M dexamethasone (Sigma-Aldrich Co. St. Louis, MO, USA). Prior to osteoblast seeding, the substrates were cleaned by three successive baths, 10-minute each, with 70% ethanol. Then, the samples were rinsed twice for 30 min in sterile-filtered Milli-Q water, air-dried and exposed to ultraviolet light in a sterile tissue culture hood, for 30 min on each side. All of the above experiments have been performed in triplicate.

### 2.3. Evaluation of Cellular Survival and Proliferation

To assess the survival capacity of MC3T3-E1 pre-osteoblasts seeded on the test materials, the LIVE/DEAD Cell Viability/Cytotoxicity Assay Kit (Molecular Probes, Eugene, OR, USA) was used. This kit is based on the simultaneous staining of viable cells which convert the non-fluorescent cell permeable dye, calcein acetoxymethyl (AM), to the green fluorescent calcein, and of the dead cells marked with ethidium homodimer-1 (EthD-1) which labels nucleic acids of membrane-compromised cells in red. This assay was performed at 1 day and 3 days post-seeding, as previously shown [40]. Briefly, the cell-populated substrates were washed with phosphate buffered saline (PBS, Life Technologies Corporation, Grand Island, NY, USA), incubated with a solution containing 2 µM calcein AM and 4 µM EthD-1 for 10 minutes in the dark, and washed again with PBS. Afterwards, they were visualized under an inverted fluorescence microscope Olympus IX71 (Olympus, Tokyo, Japan) and representative fields were captured using the Cell F image acquisition system (Version 5.0, Olympus Soft Imaging Solutions, Münster, Germany).

To quantify the viability and proliferation of the pre-osteoblasts grown in contact with the analyzed samples, the assays of the lactate dehydrogenase (LDH) release into the culture media and of the cells’ potential to reduce the water-soluble tetrazolium salt WST-8 (2-(2-methoxy-4-nitrophenyl)-3-(4-nitrophenyl)-5-(2,4-disulfophenyl)-2H-tetrazolium, monosodium salt) were conducted. More specifically, the amount of LDH released from the cytosol of dead cells as a result of materials’ cytotoxicity was measured by using “LDH-based In Vitro Toxicology Assay Kit” (Sigma-Aldrich Co. St. Louis, MO, USA) according to the manufacturer’s protocol. The optical density (OD) of the reaction product was measured at 490 nm using an automatic plate reader (FlexStation 3 Multi-Mode Microplate Reader, Molecular Devices, San Jose, CA, USA). The potential of the MC3T3-E1 pre-osteoblasts to reduce WST-8 compound to a soluble formazan was quantified in cell culture medium by using the Cell Counting Kit-8 assay (CCK-8, Sigma-Aldrich Co.St. Louis, MO, USA). For this purpose, the culture medium was discarded and then replaced with fresh culture medium containing 10% CCK-8 reagent. After 2 hours incubation step at 37 °C in a humidified 5% CO_2_ atmosphere, optical density (OD) was measured at 450 nm using an automatic plate reader (FlexStation 3 Multi-Mode Microplate Reader, Molecular Devices, San Jose, CA, USA).

### 2.4. Evaluation of Cell Adhesion and Morphology

To assess the cell adhesion and morphological features, MC3T3-E1 pre-osteoblasts in contact with the tested surfaces were fixed with a cold solution of 4% paraformaldehyde (Sigma-Aldrich Co., Steinheim, Germany) in PBS, blocked and permeabilized with a solution containing 2% bovine serum albumin (BSA, Sigma-Aldrich Co., Steinheim, Germany) and 0.1% Triton X-100 (Sigma-Aldrich Co., Steinheim, Germany) in PBS. This step was followed by an incubation with a mouse monoclonal anti-vinculin antibody (Santa Cruz Biotechnology, Dallas, TX, USA), dilution 1:50 in PBS containing 1.2% BSA, for 1 hour at room temperature. Afterwards, samples were incubated with a goat anti-mouse IgG antibody coupled with AlexaFluor 546, (Invitrogen, Eugene, OR, USA) diluted 1:200 in PBS containing 1.2% BSA, for 30 minutes in the dark. To stain the actin filaments, another incubation step with phalloidin coupled with AlexaFluor 488 (20 μg/mL, Invitrogen, Eugene, OR, USA) for 30 minutes in the dark, was performed. In a final step, the cells were incubated with 2 µg/mL 4’-6-diamidino-2-phenylindole (DAPI, Sigma-Aldrich Co., Steinheim, Germany), a specific dye for nuclei, for 10 minutes, in the dark. Each incubation step was followed by successive washes with PBS. The samples were visualized using a fluorescence microscope (Olympus IX71, Olympus, Tokyo, Japan). Representative images were captured using the Cell F acquisition system (Version 5.0, Olympus Soft Imaging Solutions, Münster, Germany).

The spread cell area and the number of focal adhesions per cell were analyzed with Image J software (Version 1.52d, National Institutes of Health, Bethesda, MD, USA). For measuring the spread area of each cell, the freehand selection tool was used. In order to quantify the number of focal adhesions, fluorescence images of vinculin staining, taken at 40×, were analyzed. In a first step, representative captured fields were transformed into grayscale images and the background was subtracted. Thereafter, the threshold was adjusted and the focal adhesions were counted using analyze particles function. 

In addition, the cellular morphology parameters such as nuclear area/cytoplasm area ratio, nuclear elongation factor and cell roundness were analyzed by following the contour of each cell manually (n = 30) using ImageJ software [41]. Thus, nuclear elongation was calculated as major/minor axis of nucleus while roundness was expressed as 4 × area/(π × major_axis^2) of cytoplasm. By definition, roundness is equal to 1 for a completely round cell.

### 2.5. Assay of Pre-Osteoblast Cell Differentiation

#### 2.5.1. Evaluation of the Intracellular Alkaline Phosphatase Activity

Alkaline phosphatase (ALP) activity, an early marker of osteoblast differentiation, was quantitatively determined in cell lysates by a method based on alkaline hydrolysis of p-nitrophenyl phosphate (pNPP), a colorless organic compound, to p-nitrophenol (pNP), a yellow compound, and inorganic phosphate. The activity of this enzyme was measured at 7 days and 14 days after cell seeding using Alkaline Phosphatase Activity Colorimetric Assay Kit (BV-K412-500, BioVision, Milpitas, CA, USA), as reported in a previous study [42]. Briefly, at the end of the incubation period, the MC3T3-E1 pre-osteoblasts grown in contact with the materials’ surfaces were lysed and centrifuged to remove the cell debris. Then, the cell lysate was mixed with pNPP solution and incubated for 60 minutes at 25 °C in the dark. The OD values of the reaction products were measured at 405 nm using an automatic plate reader (FlexStation 3 Multi-Mode Microplate Reader, Molecular Devices, San Jose, CA, USA) and related to a standard curve for ALP activity calculation. To avoid variations due to different protein concentrations, ALP activity was normalized to the corresponding protein concentration, previously determined by Bradford reaction. 

#### 2.5.2. Quantitation of the Secreted Osteopontin 

The secretion of osteopontin (OPN) was determined in the culture media after 7, 14 and 21 days of cell incubation. For this purpose, the culture media were harvested, centrifuged to remove the debris and the supernatant was used for analysis, as shown in a previous paper [42]. An enzyme-linked immunosorbent assay (ELISA) technique was performed in accordance with the manufacturer’s instructions (R&D Systems, Minneapolis, MN, USA). 

#### 2.5.3. Immunofluorescence Detection of Osteocalcin Expression

The level of the osteocalcin (OCN) expression on the materials’ surfaces was assessed at 4 weeks post-seeding by immunofluorescence microscopy. Thus, after fixing, permeabilizing and blocking the cells, as mentioned in Section 2.4, the cell-populated samples were incubated for 2 h with primary anti-osteocalcin antibody (Santa Cruz, 1:50 dilution), washed with PBS and, subsequently, incubated in the dark with goat anti-mouse IgG antibody coupled with AlexaFluor 488 (1:200, Invitrogen, Eugene, OR, USA), for 30 minutes. Finally, the nuclei were labeled with DAPI (Sigma-Aldrich Co., Steinheim, Germany) for 10 mins. The labeled samples were visualized under the fluorescence inverted microscope (Olympus IX71, Olympus, Tokyo, Japan). Representative microscopic fields were captured using the Cell F (Version 5.0, Olympus Soft Imaging Solutions, Münster, Germany) acquisition system and the mean fluorescence intensity was measured using ImageJ software (Version 1.52d, National Institutes of Health, Bethesda, MD, USA).

### 2.6. Statistical Analysis

Statistical analysis of data was performed with GraphPad Prism software (Version 6, GraphPad, San Diego, CA, USA) using one-way ANOVA/two-way ANOVA with Bonferroni’s multiple comparison tests. All values are expressed as means ± standard deviation (SD) and differences at *p* < 0.05 were considered statistically significant. 

## 3. Results and Discussions 

### 3.1. Nanotube Morphology and Characterization

**Figure 1:**
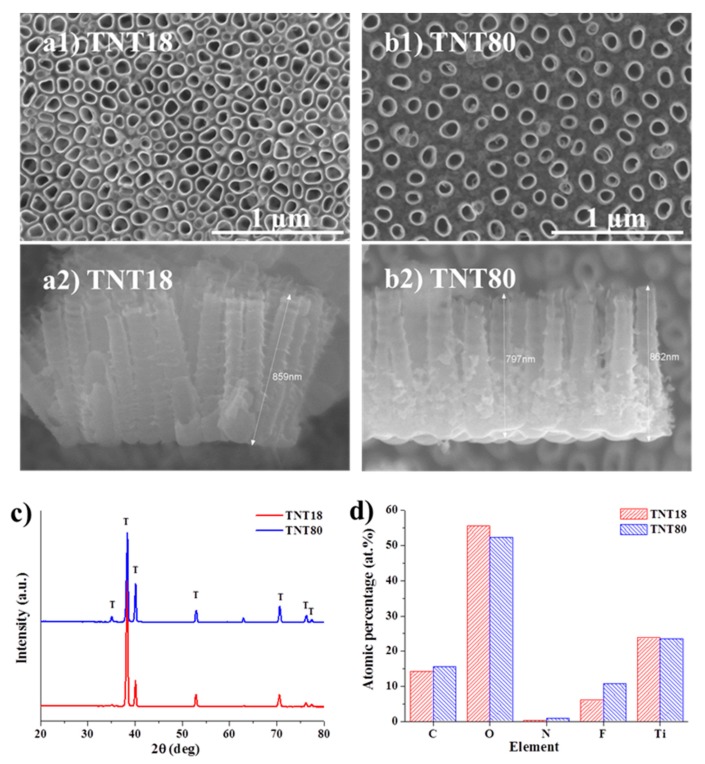
Top view and cross section SEM images of (**a1**,**a2**) TNT18, (**b1**,**b2**) TNT80. (**c**) XRD patterns of as-formed TiO_2_ nanotubes (TNT18, TNT80); (**d**) Atomic percentage data computed from X-ray photoelectron spectroscopy (XPS) measurements for the two different nanotubular structures.

As previously mentioned, TiO_2_ nanotubes grown by electrochemical anodization of Ti usually grow in a hexagonally close-packed configuration, and the tube to tube spacing observed in top view SEM images is only present at the top of the nanotubes [12,21]. Such is the case for TNT18, close packed nanotubes grown in a glycerol: water electrolyte containing NH_4_F at 20 V for 2 h [38], which have a tube diameter of ~78 nm diameter and lateral spacing of 18 nm (see also SEM images in Figure 1a).

In our previous works we have shown that the growth of spaced tubes is based on self-organization on two scales and an investigation into the critical parameters affecting the spacing of tubes obtained in DEG based electrolytes revealed that the tube-spacing originates in the initial stages of tube growth [14,43]. This spacing and the spaced nanotube morphology is controlled by the anodization conditions, e.g., electrolyte composition (water content), applied voltage and temperature [14,43].

For the present work, the anodization conditions were optimized in order to reach a similar tube diameter with that of the close packed TNT and a spacing of ~80 nm (Figure 1b). We have previously shown [43] that controlling the temperature (of the substrate) significantly affects the morphology of spaced nanotubes, namely at 30 °C spaced nanotubes are uniformly spread on the Ti substrate (high uniformity) whereas without temperature control only a local tube formation (differences between regions) is achieved for 4 h anodization experiments. Moreover, the desired nanotubular morphology should be uniform on the surface and the amount of spongy oxide (small diameter nanotubes) in between the individual spaced tubes should be minimal, just at the bottom to achieve a true individual spacing but enough to ensure the presence of standing spaced nanotubes, i.e., from ion-milled cross-section it was observed that DEG spaced nanotubes are well-embedded in a fluoride-rich layer [44,45] while anodizing at higher temperatures of 50–60 °C leads to spongy oxide free spaced nanotubes which can collapse [43]. 

The above mentioned aspects led to the optimized anodization conditions established for the spaced nanotubes used in the present study, which consist of anodization at 27 V for 4 h at 30 °C in DEG + 4 wt.% HF + 0.3 wt.% NH_4_F + 7 wt.% H_2_O, using a double anodization procedure (for more detailed information, please see experimental part). From the cross-section SEM images, it is evident that in the case of close packed TNT (TNT18), the spacing is limited to the top of nanotubes while for the spaced tubes (TNT80), the spacing is visible from top to bottom (note that the TNT layers have similar lengths, ~0.85 µm).

Both nanotubular structures are amorphous, as only peaks arising from the Ti substrate are evident in the XRD patterns (Figure 1c). Additionally, by measuring the XPS spectra and computing the atomic percentage of elements (Figure 1d), we observed no significant difference between the samples—the slightly higher fluorine content in the spaced tubes (TNT80) is also due to the electrolyte composition (as HF is used as the main source of fluorine). As the XPS surface analysis can reach up to 5–10 nm of the top surface, we have also measured the EDX of samples, 3.8 at.% F for TNT18 and 5.0 at.% F for TNT80—the percentages obtained by both XPS and EDX are in line with literature data for nanotube surfaces [21,46]. Moreover, as it was previously shown that the nanotopography of the microenvironment is a dominant factor in comparison to the crystallinity or the fluorine content in the nanotubes, with regard to cells adhesion and proliferation (e.g., endothelial cells, mesenchymal stem cells) [47], the slightly higher fluorine content at the top layer of spaced nanotubes is not expected to influence the cell culture tests.

### 3.2. Cell Survival and Proliferation

**Figure 2:**
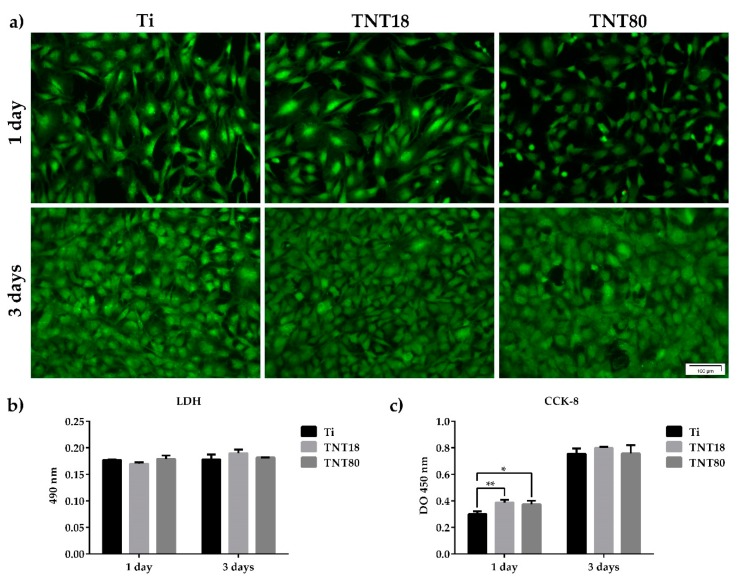
MC3T3-E1 pre-osteoblast survival and proliferation status. (**a**) Fluorescence images indicating the exclusive presence of green-labeled viable cells on all tested surfaces after staining with LIVE/DEAD Viability/Cytotoxicity kit; (**b**) The lactate dehydrogenase (LDH) levels released in the culture medium by cells grown in contact with Ti, TNT18 and TNT80 surfaces (n = 3, mean ± SD); (**c**) Pre-osteoblast proliferation capacity as evaluated by CCK-8 assay (n = 3, mean ± SD, * *p* < 0.05, ** *p* < 0.01 vs. Ti).

The cellular response to materials developed for biomedical applications is strongly influenced by their surface properties such as roughness, oxide thickness, morphology, surface energy, chemical composition, nanostructure, etc. [48,49]. Considering the importance of surface nanotopography in guiding the cell fate and behavior in terms of cell viability, proliferation and/or differentiation [7,8,9,10,11,23,24,25,26,27,28,29,30,31], a first objective of our in vitro studies was to establish the survival rate of MC3T3-E1 pre-osteoblasts grown in contact with Ti, TNT18 and TNT80 surfaces by using the LIVE/DEAD Cell Viability/Cytotoxicity Assay (Figure 2a). The fluorescent images, shown in Figure 2a, denote the presence of a cellular monolayer represented by viable green-labeled cells. No red dead cells were detected on all analyzed materials. It is also important to note that cellular population was slightly non-homogeneous in distribution and morphology on the TNT80 substrate as compared to Ti and TNT18 surfaces after 1 day of culture. However, at 3 days post-seeding, the cells became confluent on all three surfaces suggesting their increased potential to induce cell proliferation. 

Further, the absence of cytotoxicity of TNT18 and TNT80 was confirmed by estimating the activity of cytoplasmic LDH released into the culture medium by cells that have lost membrane integrity. As shown in Figure 2b, both at 1 day and 3 days post-seeding, reduced and almost equal levels of LDH activity were detected in the culture media of the cells grown in contact with all three analyzed surfaces. Therefore, TNT spacing did not induce any cytotoxicity at the studied time points, creating a favorable environment for the MC3T3-E1 pre-osteoblast growth.

These observations are also supported by the results of the CCK-8 test—an assay used for quantifying the number of viable metabolically active cells. Thus, as noted in Figure 2c, the number of pre-osteoblasts grown in contact with Ti, TNT18 and TNT80 surfaces showed a time-dependent increase from 1 day to 3 days-incubation period. Moreover, after 1 day of culture the nanotubular surfaces exhibited a higher potential to sustain cell proliferation in comparison with the flat Ti surface. However, at 3 days post-seeding a similar number of cells was identified on all analyzed materials. These results are not surprising as a previous study approaching the behavior of MC3T3-E1 pre-osteoblasts in contact with large diameter anatase titania nanotubes (70–120 nm) exhibited stimulatory effects on the cell proliferation rates at early culture stage [28]. 

Overall, it might be inferred that both analyzed nanostructured surfaces have the potential to support cell survival and proliferation. They induced a differential stimulation of cell proliferation at 24 h post-seeding (*p* < 0.05 and *p* < 0.01 for TNT80 and TNT18, respectively) but, after 72 h of culture similar OD450 values were obtained for all studied surfaces.

### 3.3. Cell Adhesion and Morphological Features

**Figure 3:**
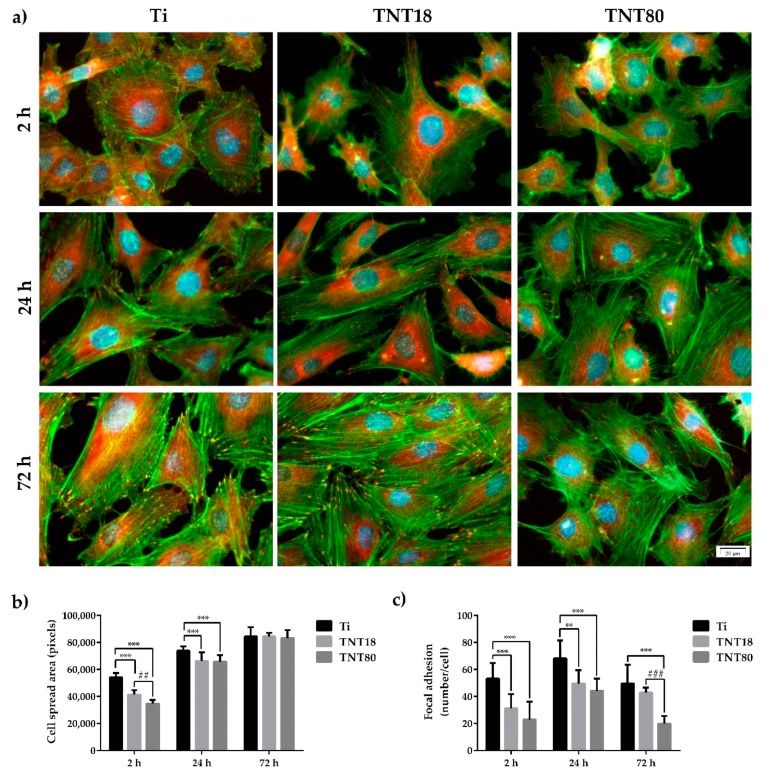
Cell adhesion, spreading and morphology at 2 h, 24 h and 72 h post-seeding. (**a**) Fluorescence micrographs used to assess adhesion and morphological characteristics of the MC3T3-E1 pre-osteoblasts maintained in contact with Ti, TNT18 and TNT80 surfaces. The cytoskeletal actin filaments were stained with phalloidin coupled with Alexa Fluor-488 (green); vinculin was labeled with anti-vinculin primary antibody and secondary antibody coupled with Alexa Fluor-546 (red); the nuclei were stained with DAPI (blue); (**b**) Quantitative analysis of cell spread area. Results are presented as means ± SD (n = 10 cells, *** *p* < 0.001 compared with Ti; ## *p* < 0.01 compared with TNT18); (**c**) Quantification of focal adhesions. Results are presented as means ± SD (n = 10 cells, ** *p* < 0.01, *** *p* < 0.001 compared with Ti; ### *p* < 0.001 compared with TNT18).

Cellular adhesion to a surface is another decisive factor in determining the biocompatibility of a biomaterial. Information on the adhesion and morphology of the MC3T3-E1 pre-osteoblasts were obtained by fluorescence microscopy after labeling of actin cytoskeleton and vinculin (Figure 3a), the last one being a key protein in focal adhesions that can stabilize and modulate the dynamics of cell adhesions [50,51]. 

Accumulating data showed that cells can sense nanometer-scale variations in the average spacing of integrin ligands [52,53], and that the interactions mediated by these receptors are essential for providing information necessary for numerous adhesion-dependent cell functions, such as cell proliferation, differentiation and survival [54,55]. For example, ordered patterns of integrin ligands with a lateral spacing larger than 73 nm limited the focal adhesion (FA) formation and cell spreading while interdot distances of ≤58 nm allowed efficient FA formation and actin stress fibers assembly, and the cells adopted a well spread morphology [52,56]. In another study by Lee et al. [57] it was demonstrated that the variation in the nanoscale spacing of Arginine-Glycine-Aspartic Acid (RGD) ligands in alginate gels influenced adhesion, proliferation, and differentiation capacities of MC3T3-E1 pre-osteoblasts, where a decrease in the RGD island spacing from 78 to 36 nm induced an enhancement of cell proliferation rates and osteocalcin secretion. However, the threshold values mentioned above cannot be generalized owing to the strong dependence of cell behavior on the substrate properties [58]. 

In the present study, the images obtained after 2 h, 24 h and 72 h of cell culture revealed differences in pre-osteoblast morphology, actin cytoskeleton organization and distribution of vinculin between the analyzed surfaces (Figure 3a). Thus, at 2 h post-seeding on the flat Ti surface, the cells displayed spread morphology and larger dimensions (Figure 3b), and thin stress fibers throughout the cell body more numerous than in pre-osteoblasts grown in contact with the investigated nanotubular surfaces. Moreover, the presence of a higher number of vinculin positive signals on this surface, predominantly localized at the cell periphery, suggests the formation of multiple focal complexes (Figure 3c). On the contrary, the pre-osteoblasts grown on TNT18 and TNT80 surfaces adopted mostly a dendritic morphology with numerous cytoplasmic projections and significantly less vinculin-rich focal contact points at their extremities (Figure 3a) with an average of 31.2 and, respectively, 22.9 compared to 53.1 (Figure 3c). At the same time, the degree of cell spreading was lower than on the Ti surface (Figure 3b). This behavior could be ascribed to the limited surface area for cell attachment on the top wall surface owing to the large inner nanotube diameter and spacing gap between nanotubes. The more spacing gap between nanotubes, the lower pre-osteoblast spreading was noticed.

After an incubation period of 24 h, the MC3T3-E1 pre-osteoblasts still exhibited distinct cell morphologies on the different surfaces. For example, the cells grown on the nanotubular surfaces were more elongated and possessed more cellular protrusions as compared with the flat Ti surface. Furthermore, the most obvious and numerous focal adhesions were expressed on the Ti substrate, revealing a progressive decrease in the following order Ti > TNT18 > TNT80 (Figure 3a,c). This finding is in line with the results reported by Park et al. [7] showing the formation of less focal contacts on larger titania nanotubes (≥70 nm diameter) than on the flat Ti substrate while more focal contacts were visible on smaller nanotubes (≤30 nm diameter). Likewise, well defined bright green-labeled actin filaments, thinner on the nanotubular surfaces, mostly oriented in a parallel direction along the cell body and within the cellular protrusions are visible (Figure 3a). Compared with the flat control surface, the pre-osteoblast cells displayed smaller average cell areas on both TNT18 and TNT80 (Figure 3b) but the differences between the nanotubular surfaces were reduced with time. 

**Figure 4:**
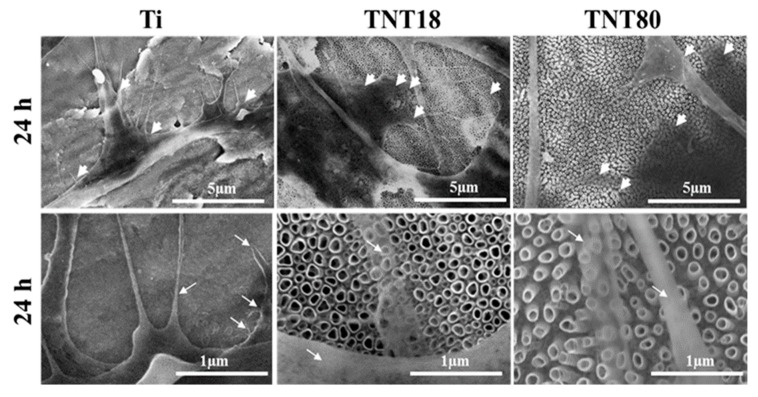
SEM images of the MC3T3-E1 pre-osteoblasts maintained in contact with Ti, TNT18 and TNT80 surfaces for 24 h. The white arrowheads show the location of cellular extensions; filopodia are indicated with white arrows.

Additionally, the scanning electron microscopy (SEM) micrographs of cells incubated for 2 h and 24 h on the analyzed surfaces, from which selected SEM images for the 24 h time incubation point are shown in Figure 4, confirmed the above morphological observations by fluorescence microscopy. Thus, besides the surface features of the three investigated materials, spread MC3T3-E1 pre-osteoblasts displaying different morphological features and cellular extensions, in the form of lamellipodia and filopodia, can be distinguished (Figure 4). Noticeable, more pronounced protrusion of filopodia with significantly longer and wider configuration, spread across the pores of both analyzed nanotubular arrays, is visible. An important characteristic of some of these filopodial extensions is their transparency, a feature that has previously been shown to be typical for the cells attached to the large diameter nanotubes [7].

**Figure 5:**
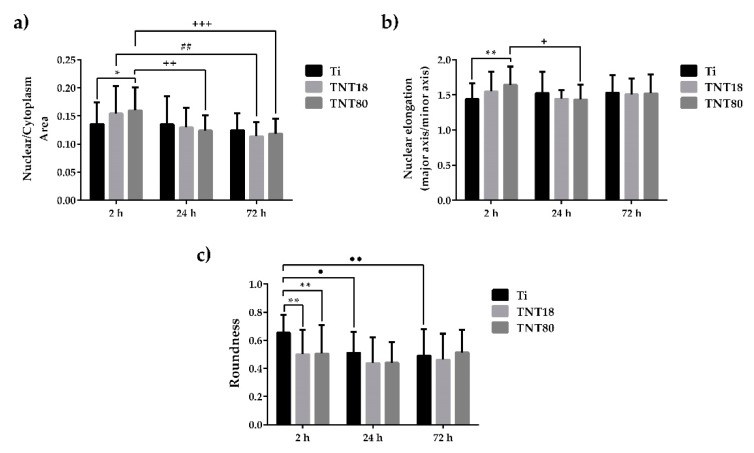
Cellular morphology parameters evaluated at 2 h, 24 h and 72 h post-seeding. (**a**) Quantification of Nuclear-Cytoplasmic area ratio. Results are presented as means ± SD (n = 30 cells, * *p* < 0.05 compared with Ti; ## *p* < 0.01 between TNT18 at 24 h vs. 72 h; ++ *p* < 0.01, +++ *p* < 0.001 between TNT80 at 2 h vs. 24 h, and 2h. vs. 72 h, respectively); (**b**) Nuclear elongation measurement calculated as the ratio between major axis and minor axis. Results are presented as means ± SD (n = 30 cells, ** *p* < 0.01 compared with Ti; + *p* < 0.05 between respective groups at 2 h vs. 24 h); (**c**) Quantification of cell roundness. Results are expressed as means ± SD (n = 30 cells, ** *p* < 0.01 compared with Ti; ● *p* < 0.05, ●● *p* < 0.01 comparation between Ti at 2 h vs. 24 h, and 2h vs. 72 h, respectively).

Actin/vinculin immunofluorescence analysis has also been performed at 72 h post-seeding. As shown in Figure 3, at this point in time both nanotubular arrays influence the cells’ shape and the organization of the actin cytoskeleton (Figure 3a), as well as the pattern and number of focal adhesions (Figure 3a,c). Thus, the adherent cells on the TNT18 surface exhibited polygonal or spindle-shaped osteoblast-like morphologies similar to the pre-osteoblasts grown in contact with the flat Ti surface but on the TNT80 substrate they adopted mixed shapes, either a less-broad cobblestone-like or spindle-shaped morphology (Figure 3a). Furthermore, well-defined actin stress fibers oriented in a parallel fashion along the main cellular axis and vinculin immunoreactive sites at their termini are visible on the flat Ti and TNT18 surfaces. On the contrary, MC3T3-E1 cells grown in direct contact with the TNT80 substrate showed mainly a branched actin filament network as well as more intercellular connections established at the level of the filopodial and lamellipodial protrusions. Likewise, they exhibited a significantly lower number of focal adhesions than on Ti and TNT18 surfaces (Figure 3c). We speculate that the larger spacing gap may provide less chance for cells to form integrin clustering leading to focal contact formation compared to less spaced, dense TNT with the same inner diameter (Figure 3 and Figure 4). Importantly, the vinculin signals exhibited a punctiform pattern on the flat Ti surface whereas on both studied titania nanotubes they appeared to be elongated, suggesting acquisition by these cells of a more migratory phenotype [59,60]. This finding is consistent with previously reported studies showing that various human cell types display enhanced motility on nanotopographies compared with flat surfaces [7,61,62]. It is noteworthy that the vinculin recruitment to the focal adhesion sites has been shown to correlate directly with the traction force applied on the same focal adhesion [63]. It reinforces focal adhesions by crosslinking actin filaments to a large cytoskeletal molecule, talin, a critical step in cell mechanics connecting the cell to its substrate [64,65]. Actually, vinculin conveys force inside-out by increasing integrin–talin complexes via the head domain, while its tail domain is needed to propagate force to the cytoskeletal actin. To note that the assembly of focal adhesions is affected not only by internally-generated forces exerted on them [66], but also by the physical state and mechanical properties of the external cellular environment [56]. Of particular interest is the modulation of the number, arrangement, and size of focal adhesions, redistribution of cyto-and nucleo-skeletal components, as well as of cell and nuclear morphologies by nanotopography [67,68,69,70]. In fact, nanostructured surfaces evoke architectural rearrangements that activate, through focal adhesions, the signaling cascades leading to indirect downstream effects on gene expression and induce mechanical changes in the cell that involve physical pulling of the cytoskeleton on the nucleus. These induce changes in gene transcription by imposing mechanical forces on nuclear components. Considering these potential mechanotransductive effects elicited by nanotopographical surfaces on cell structural components and the above results showing the modulation of the pre-osteoblast behavior in terms of cell adhesion and morphology, cytoskeleton organization, cellular expansion, and focal adhesion patterns by lateral spacing of titania nanotubes, we further evaluated the next cellular spreading parameters: nuclear area/cytoplasm area ratio, nuclear elongation factor and cell roundness (Figure 5). These parameters can provide information about the extent of the cellular response to traction forces coming from the underlying substrates and of the cellular forces exerted on them [41]. In this way the cells can probe the rigidity of the extracellular environment, develop focal adhesions, trigger signaling, and remodel the extracellular matrix forces. As the results show, cell stretching and nuclear elongation were distinguishable only in initial phase of cultivation (2 h) between groups. This result is in agreement with our results showing cell spreading area. Considering that focal contacts on TNT80 were constantly less detectable throughout observation period (2 h–72 h), focal contact formation certainly via cell-substrate sensing mechanism including integrin signaling might be continuously controlled by TNT topographic differences. This result seems to be in good accordance with previous studies showing that larger lateral gap provides less focal contact formation.

### 3.4. Pre-osteoblast Cell Differentiation

Generally, nanostructures have been reported to support the osteogenic differentiation of stem cells and osteoblasts [24,71,72]. In this study, the expression of the bone cell-specific markers such as ALP, OPN and OCN was measured.

**Figure 6:**
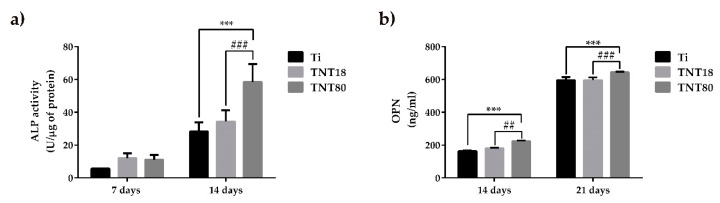
Evaluation of alkaline phosphatase (ALP) and osteopontin (OPN) expression. (**a**) The expression level of intracellular ALP activity of osteoblasts grown on Ti, TNT18 and TNT80 surface at 7 and 14 days in the presence of osteogenic differentiation media (n = 3, mean ± SD, *** *p* < 0.001 compared with Ti; ### *p* < 0.001 compared with TNT18); (**b**) The expression level of osteopontin released into the culture medium by osteoblasts cultivated on the tested surfaces for 14 and 21 days in the presence of osteogenic medium (n = 3, mean ± SD, *** *p* < 0.001 compared with Ti; ## *p* < 0.01, ### *p* < 0.001 compared with TNT18).

ALP is a ubiquitous membrane-bound homodimeric metalloenzyme that catalyzes the hydrolysis of phospho-monoesters, releasing inorganic phosphate (Pi) and alcohol, and is one of the most commonly used biochemical markers to assess the osteoblast activity [73]. It appears that the mechanism of action of this enzyme consists both in increasing the local concentration of inorganic phosphate, a mineralization promoter, and in reducing the extracellular concentration of pyrophosphate, a mineralization inhibitor. In the present study, intracellular ALP activity was quantified at 7-days and 14-days post-seeding in order to estimate the ability of tested materials to induce bone mineralization. As it can be seen in Figure 6a, after 7 days of cell incubation under osteogenic conditions, the nanotube coated surfaces (TNT18 and TNT80) induced an increase in ALP activity by ~50% compared to the Ti surface. It is also noted that at 14 days post-seeding, ALP activity recorded increased values on all analyzed materials. Furthermore, at this time point, the differences in the expression of ALP activity by the pre-osteoblasts grown on all three surfaces were more obvious than at 7 days of cell incubation. Specifically, both nanotubular surfaces exhibited higher levels of ALP activity in comparison with the flat Ti surface. This finding is not surprising since, overall, nanotubular TiO_2_ surfaces are well known for their ability to enhance ALP activity [27,31,74]. However, in the present study, this enhancement was significant in the case of the pre-osteoblasts grown on TNT80 substrate. Furthermore, ALP activity in the lysates of these cells recorded a significant increase when compared with intracellular ALP activity exhibited by the cells grown in contact with TNT18 surface.

In order to get a more complete picture of the ability of the analyzed surfaces to induce the early cell differentiation, the concentration of OPN secreted in the culture medium by MC3T3-E1 cells grown on Ti, TNT18 and TNT80 was determined by ELISA technique at 14 days and 21 days post-seeding (Figure 6b). Osteopontin is a highly phosphorylated glycoprotein that strongly links to extracellular matrix non-collagen proteins, and exhibits multiple biological functions [75]. For instance, OPN in the osseous tissue is released from osteoblasts and osteoclasts eliciting three major functions during biomineralization phase of bone structuring including modulation of bone cells adhesion, modulation of osteoclast function, and modulation of matrix mineralization, as well. The results obtained in the present study showed a time-dependent increase in OPN synthesis and extracellular release in culture media maintained in contact with all analyzed substrates. It is noteworthy that TNT80 elicited a stronger effect in inducing OPN secretion and, implicitly, pre-osteoblast differentiation in comparison with TNT18 and Ti surfaces at both incubation time points.

The next objective of our study was to quantify the level of expression for the most abundant non-collagenous bone matrix protein, OCN, in MC3T3-E1 pre-osteoblasts grown in direct contact with the three analyzed surfaces. This protein is often studied as a late marker for bone formation, playing the role of a regulator in bone mineralization and bone turnover [76,77]. However, it can be stated that the exact role of OCN in bone is still incompletely understood, although several lines of evidence proved that OCN enhances bone formation. For example, it was shown that OCN increases the adhesion of osteoblast-like cells on biocement [78]. In addition, Rammelt et al. demonstrated its potential to enhance the appearance of active osteoblasts and bone healing around hydroxyapatite/collagen composites [79]. 

**Figure 7:**
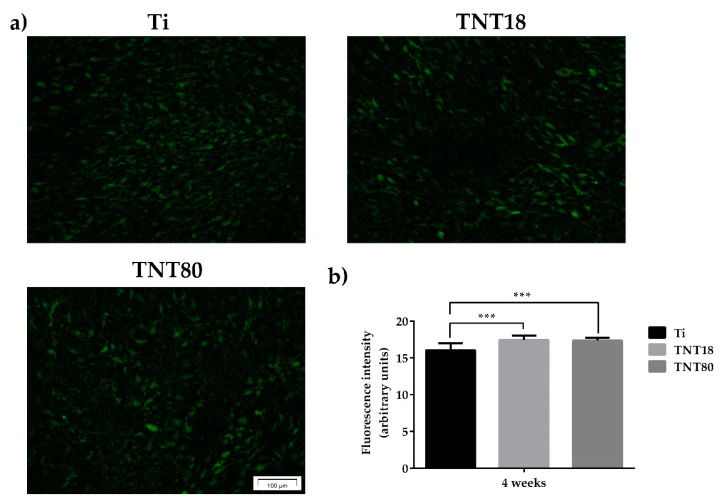
Evaluation of OCN expression. (**a**) Fluorescent images that highlight osteocalcin expression in MC3T3-E1 osteoblasts grown on Ti, TNT18, TNT80 surfaces for 4 weeks in the presence of osteogenic differentiation media using anti-osteocalcin (green) specific antibody; (**b**) Measurement of fluorescence intensity using Image J software (n = 20, mean ± SD, *** *p* < 0.001).

As shown in Figure 7a, immunofluorescence detection of OCN expression in MC3T3-E1 pre-osteoblasts grown for 4 weeks in contact with Ti, TNT18 and TNT80 materials, under osteogenic conditions, denotes quite a similar staining pattern of this protein on their surfaces. However, the quantification of the OCN fluorescence intensity (Figure 7b) indicates that both nanotubular surfaces induced a statistically significant increase in the expression of this osteoblast differentiation marker when compared to flat Ti surface.

The above-mentioned results are in good agreement with previous studies on MSC [24] and pre-osteoblast [27,28,80] showing that large diameter (70–100 nm) TiO_2_ nanotubes strongly induced osteogenic differentiation when compared to smaller diameter nanotubes. Hence, taken together, these experimental data emphasize the ability of the analyzed nanotubular surfaces, mainly TNT80, to enhance the induction of osteoblast differentiation. It is becoming increasingly clear that nanotopograhy represents a viable strategy to modulate cell differentiation and that the cell function is highly regulated by mechanotransduction [81,82]. Taking into account the results of this study, we assume that one of the mechanisms responsible for the differential osteogenic response of the MC3T3-E1 pre-osteoblasts is driven by the mechanotransductive signals induced by the analyzed surfaces. This assumption is supported by a recent study performed by Zhang et al. [80] who investigated the intracellular mechanisms involved in stimulation of the osteogenic differentiation of MC3T3-E1 cells by large diameter titania nanotubes (LTNTs; 90 nm inner diameter) in comparison to small diameter nanotubes (STNTs; 30 nm inner diameter) and flat Ti surface. The Real-time PCR analysis showed that LTNTs elicit increased gene expression of the bone differentiation markers, Runt-related transcription factor 2 (Runx2) and osterix (Osx), when compared with cells in contact with flat Ti surfaces. This finding has also been confirmed by histological analysis performed on the regeneration bone tissue after implantation into rat tibiae showing that titania nanotubes, mainly LTNTs, induced better implant osseointegration. To clarify the underlying mechanisms of this differential osteogenic response provoked by the analyzed surfaces, the expression levels of focal adhesion kinase, both total (FAK) and activated (pY397-FAK), as well as FAK recruitment to focal adhesions have also been investigated. The results demonstrated that when compared to flat Ti substrate, both nanotubular surfaces, more significantly LTNTs diminished FAK activity and its recruitment to focal adhesions. As a result, a reduction in the activity of the Ras homolog gene family member A (RhoA), a small GTPase able to sense and respond to mechanical cues, occurred. RhoA and FAK interact together in order to perceive the mechanical stimuli and regulate cell differentiation [83]. The alteration of the FAK/Rho signaling was followed by the export in cytosol of the Yes-associated protein (YAP), which has been shown to be implicated in transmission of mechanical signals to the nucleus, and activation of the bone differentiation marker Runt-related transcription factor 2 (Runx2). This export reduced the YAP/Runx2 binding probability leading to the Runx2 activation and initiation of the osteogenesis on nanotubes. Considering the stimulatory effects of these larger lateral gaps on osteogenic induction, our results may indicate that larger spacing gaps play a role in enhancing osteogenic induction of pre-osteoblastic cells in addition to the effect of large nanotube diameter.

We do not rule out other nanotopography-mediated signal transduction pathways or the above presented mechanism that could be responsible for the modulation of pre-osteoblast behavior by lateral nanotube spacing. Further research will be necessary to confirm these assumptions and elucidate the underlying mechanisms.

## 4. Conclusions

Our results provide some insight on the effects of titania nanotube spacing on osteoblast cell functions in vitro. Both nanotubular structures, closed packed nanotubes with tube to tube spacing at the top of 18 nm and spaced nanotubes with tube to tube spacing of 80 nm, were found to sustain cell viability and proliferation to almost a similar extent as the flat Ti substrate. Furthermore, the data obtained demonstrate that spaced nanotubes (tube to tube spacing of 80 nm) influence other aspects of cell behavior including cell adhesion and morphology, cytoskeleton organization and focal adhesion patterns, as well as osteogenic differentiation. They have been proved to elicit beneficial effects on the pre-osteoblast osteogenic differentiation, as demonstrated by the increased ALP activity, and osteopontin and osteocalcin protein expression. Overall, the study provides a new variable worthy of investigation in designing and optimizing titanium-based materials as platforms for bone regeneration and drug delivery.
